# Research feasibility and ethics in Scottish new-born blood spot archive.

**DOI:** 10.23889/ijpds.v7i3.2027

**Published:** 2022-08-25

**Authors:** Sarah Cunningham-Burley, Daniel L McCartney, Archie Campbell, Robin Flaig, Clare El Orange, Carol Porteous, Mhairi Aitken, Ciaran Mulholland, Sara Davidson, Selena M McCafferty, Lee Murphy, Nicola Wrobel, Sarah McCafferty, Karen Wallace, David Stclair, Shona Kerr, Caroline Hayward, Andrew M McIntosh, Cathie Sudlow, Riccardo E Marioni, Jill Pell, Zosia Miedzybrodzka, David J Porteous

**Affiliations:** 1 University of Edinburgh; 2 NHS GGC Biorepository; 3 Ipsos MORI Scotland; 4 University of Aberdeen; 5 University of Glasgow

## Objectives

There were two objectives to this study: 1) to gauge public opinion on the use of Guthrie card-derived blood samples for epidemiological and biological research; and 2) to evaluate the feasibility of recovering meaningful molecular data from these samples.

## Approach

To address the first objective, a 2-day Citizens’ Jury was conducted in partnership with Ipsos MORI, comprising a diverse adult sample in terms of age, sex, working status and social grade (n=20). Jurors were asked whether research access to Guthrie card blood tests would be in the public interest. To address the second objective, DNA methylation (DNAm) was profiled from samples from 58 Generation Scotland participants, whose Guthrie cards had been stored from birth for between 32 and 38 years. Analyses were performed on Guthrie DNAm samples to determine whether previously-reported associations with perinatal maternal smoking behaviours were detectable.

## Results

The Citizens’ Jury yielded an overall positive response towards data sharing for health research. Concerns were raised about data protection and security, control and oversight, and commercial use. The overall verdict was that access to Guthrie card data would be in the public interest, conditional on the purpose of the research, regulated access procedures, ethical oversight and provision of opportunities for participants to opt out.

DNAm detection rates from Guthrie samples were lower than from samples stored in tubes. However, it was possible to confirm linkage to the correct individuals in Generation Scotland using DNAm-derived estimates of genotype and sex. A significant association was observed between a DNAm-based score for smoking and perinatal maternal smoking status derived from the baseline Generation Scotland questionnaire.

## Conclusion

We showed that: 1) public support exists for using Guthrie samples in research, conditional on certain safeguards; 2) DNAm can be profiled from cards stored for up to 38 years and can predict maternal smoking behaviour. Guthrie cards are a potentially valuable resource for epidemiological studies and predicting health outcomes.

